# Burnout: need help?

**DOI:** 10.1186/1745-6673-3-32

**Published:** 2008-12-05

**Authors:** Betul Gulalp, Ozgur Karcioglu, Azade Sari, Zikret Koseoglu

**Affiliations:** 1 Department of Emergency Medicine, School Of Medicine, Baskent University, Adana, Turkey; 2 Department of Emergency Medicine, Bakirkoy Dr. Sadi Konuk Research and Training Hospital, Bakirkoy, Istanbul, Turkey; 3 Department of Emergency Medicine, Cukurova State Hospital, Adana, Turkey; 4 Department of Emergency Medicine, Numune Research and Training Hospital, Adana, Turkey

## Abstract

**Background:**

Burnout syndrome is a psychological situation induced with working, especially in high-risk parts of the hospitals that affects the physical and mental conditions of the staff. The aim is to identify the characteristics of the staff related to Burnout Syndrome in the Emergency Department (ED).

**Methods:**

The study includes the Maslach Burnout Inventory and other new individual research questions. The responders were the volunteers and comprised physicians, nurses, nurses' aides from EDs of all urban state hospitals of Adana (43.3%). Burnout scores were analyzed with regard to individual characteristics; supplementary work, marital status, the number of children, occupation, salary, career satisfaction, satisfaction in private life. Mann-Whitney U test and Kruskall-Wallis test were performed using SPSS 15.00.

**Results:**

There were no relation between Burnout scores and supplementary work, marital status, number of children, occupation, salary, private life satisfaction, except for career satisfaction.

**Conclusion:**

Presence and severity of Burnout syndrome were linked to career satisfaction without personal features and salaries. All branches of healthcare occupations in ED seem to have been affected by Burnout Syndrome similarly.

## Background

Burnout is a syndrome explained as serious emotional depletion and behaviour with a poor adaptation at work due to prolonged occupational stress [1]. It has three principal components of emotional exhaustion, depersonalization, and diminished feelings of personal accomplishment. Emotional exhaustion is characterized by personal feebleness. Lack of personal accomplishment indicates the failure to achieve the individual aims and depersonalisation is listlessness while working [1-3]. It is a work-related syndrome and it is most likely to occur in Emergency Departments (ED) that generally manage life threatening conditions [3]. The staff have high stress due to a myriad of reasons; including overcrowded departments, difficulty of cases, work schedules, disorganized ED, deficient number of staff [4]. Burnout is accused to be the main cause of impotent motivation. The specific risk factors for ED staff have not been clearly established. The aim of this study is to identify the relationship between Burnout Syndrome in ED and individual characteristics of the staff.

## Methods

Maslach Burnout individual-based study was conducted in three state hospitals in one of the biggest cities of Southern part of Turkey, Adana, between May 1 and 31, 2006. The inventories were given to voluntary participants on their shifts to all groups after the information was described. All healthcare workers in the ED were asked to participate in the study which included a total of 77 emergency doctors, 76 emergency nurses, 55 nurses' aide. The self-report forms were advised to be filled alone during the shifts and were collected by the directors of the departments. They were received via closed, without revealing the name or any personal identification clue on the same day. In this descriptive study, Burnout variables were studied with a total of 22 questions of Turkish version of Maslach Burnout inventory [5,6]. There were 3 subgroups that include 9 questions about emotional exhaustion, 5 questions were relevant to depersonalization and 8 questions involved in personal accomplishment. The participant gave a score to the questions between 0 (never) and 4 (always). The subjects were asked to respond to specific questions relevant to eight individual issues: supplementary work, marital status, number of children, drug and alcohol use, cigarette smoking (≥10/day), individual suggestions about regular stress, management training and professional psychiatric support of the staff against Burnout, occupation, salary, career satisfaction and finally, private life satisfaction and family relations. The career satisfaction of working in ED in every detail was asked to the volunteers of the study. Private life satisfaction included the general evaluation of all kinds of private life and family relations by the participant. Supplementary work meant extra working in a private health center in addition to 160 hours/month for a regular job in ED of the state hospital. As the acquired data were non-parametric, comparisons between two groups were performed using Mann Whitney U test and comparisons among three groups were analyzed with Kruskal Wallis test, SPSS 15. The differences were accepted as significant when the p value was below 0.05.

## Results

The study included only EDs of the regional state hospitals of the city, each with a daily volume of 600 to 800 patients. The participants were the physicians, nurses and nurses'aide and have similar shift periods as 12 hours (min 8-max 16). There were not any influence from company staff regarding negative effects while participants were answering the questions. All of the three hospital are currently up-dated, modernised and managed with positive support to the staff. There were not any public event which may have markedly affected the overall workload of the hospitals during the study. The study included 90 (43.3%) staff who had consented for recruitment from the EDs of three state hospitals in Adana in May 2006. In total, 38 (49.3%) emergency physicians, 40 (52.6%) nurses, and 12 (21.8%) nurses' aide were enrolled. The mean exhaustion score was 19.1 ± 9.1(0–36), while personal accomplishment score was 22.3 ± 5.9(8–32), and depersonalization score was 7.8 ± 4.7(1–18). Tables 1 to 4 delineate Burnout scores regarding supplementary work, married status, number of children, career satisfaction, respectively. There were not any significant differences between physicians, nurses and nurse'aides according to the occupation for emotional exhaustion, personal accomplishment, depersonalization (p values 0.3, 0.2, 0.6, respectively). The significant difference values according to salaries for emotional exhaustion, personal accomplishment and depersonalization were 0.08, 0.5, 0.5, respectively. The significant difference values according to private life satisfaction for emotional exhaustion, personal accomplishment, depersonalization were 0.11, 0.6, 0.3, respectively. Beside them, 53% of the staff refused to work in ED (n = 48). Forty-one percent of the respondents were cigarette smoker (n = 37). The percentage of unanswered questions about drug and/or alcohol consumption were 22.2% (n = 20), and 77.8% of the answers were negative. Eigthy-four percent of the staff accepted the regular stress management training and physchological support against Burnout Syndrome (n = 76), whereas 16% refused the suggestion (n = 14).

**Table 1:** The relation between Burnout scores according to supplementary work are shown.

		N	Mean	Median	ss	Min	Max	Line median.	Mann-Whitney U	p
Emotional exhaustion	Yes	9	18,3	23	10,8	4	35	44,1	351,5	0,861
			
	No	81	19,2	20	9,0	0	36	45,7		

Personal accomplishment	Yes	9	24,0	25	5,5	15	31	52,6	300,5	0,388
			
	No	81	22,2	23	6,0	8	36	44,7		

Depersonalization	Yes	9	7,0	8	3,7	0	11	42,3	336	0,701
			
	No	81	7,9	7	4,9	0	18	45,9		

**Table 2:** The relation between Burnout scores and marital status are demonstrated.

		N	Mean	Median	ss	Min	Max	Line median	Kruskall-Wallis H	SD	p
Emotional exhaustion	M	65	19,0	20	9,3	0	36	45,2	0,593	2	0,743
				
	W	7	17,1	22	8,8	4	27	40,0			
				
	U	18	20,5	21	9,0	5	36	48,7			

Personal accomplishment	M	65	22,8	23	6,0	11	36	47,0	0,751	2	0,686
				
	W	7	22,0	23	4,7	18	31	41,6			
				
	U	18	21,1	21,5	6,2	8	31	41,7			

Depersonalization	M	65	7,5	7	4,5	0	17	44,2	0,915	2	0,632
				
	W	7	7,7	5	5,8	0	17	43,9			
				
	U	18	9,0	7,5	5,2	1	18	50,8			

M = Married, W = Widowed, U = Unmarried.

**Table 3:** The relation between Burnout scores and the number of children are shown.

		N	Mean	Median	ss	Min	Max	Line Medium	Kruskall-Wallis	SD	p
Emotional exhaustion	0	11	19,9	22	6,4	7	28	38,7	0,427	2	0,808
				
	1	28	19,1	20,5	9,2	0	35	37,7			
				
	2+ children	33	18,2	19	10,1	4	36	34,8			

Personal accomplishment	0	11	21,9	21	5,6	11	32	34,5	1,055	2	0,590
				
	1	28	22,1	23	5,1	15	32	34,1			
				
	2+children	33	23,5	24	6,6	12	36	39,2			

Depersonalization	0	11	6,6	8	3,6	0	12	33,9	0,228	2	0,892
				
	1	28	7,7	6	4,7	0	17	37,4			
				
	2+children	33	7,6	6	5,0	0	17	36,6			

**Table 4:** The relation between Burnout scores and career satisfaction are shown.

		N	Mean	Median	ss	Minimum	Maximum	Line Medium.	Mann-Whitney U	P
Emotional exhaustion	Yes	38	15,1	14,5	8,1	4	35	23,1	137	0,000
			
	No	22	26,0	25	6,6	13	36	43,3		

Personal accomplisment	Yes	38	24,2	24,5	5,7	12	36	35,2	241	0,006
			
	No	22	19,5	19	5,9	8	32	22,5		

Depersonalization	Yes	38	6,1	6	4,3	0	16	24,5	190	0,000
			
	No	22	10,3	10	4,0	2	16	40,9		

## Discussion

Emergency staff are seriously under stress as various patients with frequent shifts in a high risk of violence directed towards them. In the EDs of state hospitals aproximately 600 to 800 patients are managed and disposed in 24 hours. There is a twelve-hour shift system in the recruited EDs, since longer working hours can be a serious wearing factor [7]. Silva et al reported that the rate of emotional exhaustion was 23%, depersonalization was 36.1%, personal accomplishment was 33.3% [8]. Gonzales et al mentioned the rates were as 59.7% for emotional exhaustion, 36.1% for depersonalization, 31.2% for personal accomplishment as in their study [9]. We found that 53% of respondents demonstrated emotional exhaustion, 39% had depersonalization, and 46% reported lack of personal accomplishment. In this study the ratio of lack of personal accomplishment compared to the literature can be attributed to local circumstances of this region. Keller and Koening reported that 53% of emergency physicians planned to work at least ten years longer, and only 24% of them foretold working for twenty years [10]. Kalemoglu mentioned that the acquiescence to work in ED for two years was 36%, more than three years was 14% [11]. In this study 53% of the staff refused to work in ED anymore. The recent reports attributed this phenomenon to overcrowdedness in ED, disorganization, understaffing, agressive behaviors of the patients, and finally, low income with personal and environmental characteristics. Howsoever, beside the stress in department, private life satisfaction with family relations can be affected on Burnout Syndrome for ED staff [4]. Kalemoglu et al and Whitley et al [11,12] reported that single ED physicians showed depression findings more than married ones. Taycan found that emotional exhaustion in singles was more common than that in married ones [13]. However, in our study there were no relations between Burnout scores and having supplementary work, marital status, number of children, private life satisfaction. These demonstrated that the other factors as working schedules, organization, relations within the team, behavioral characters of the public of the region could be the stronger predictive factors for Burnout of the ED staff due to career satisfaction and clarify with subsequent studies. Although emergency staff were not aware of Burnout before this study (n = 90, 100 %), there was a high percentage of acceptance about regular professional psychiatric support and training (n = 76, 84.4%). In a study, substance addiction was found to be more common among the residents in the ED when compared to the other departments [14]. In the present study, some of the participants refused to answer the question about using any psychiatric drugs and/or alcohol regularly (n = 20, 22.2%), and it was deemed an unexpected result. Five of them (5.5%) reported to use alcohol, while two others were using antidepressant drugs regularly. There were some limitations in this study. The study included nearly half of the staff of EDs. The subscores range were wide as the experiences in ED of the staff could be different. The report comprised only physicians, nurses and nurses' aide; excluding security, secretary and other ED staff.

## Conclusion

This study reported that individual properties of staff and even salaries were not related with Burnout. New studies could focus on career satisfaction probably with organization and management methods on Burnout Syndrome to develop new strategies against Burnout Syndrome in ED.

## Competing interests

The authors declare that they have no competing interests.

## Authors' contributions

BG designed the study, collected data, carried out the statics and wrote the manuscript, OK reviewed and edited the manuscript, AS and ZK conducted the research and collected individual data in their institutions. All authors read and approved the final manuscript.
